# Pharmacokinetic-dynamic relationship of cisplatin in vitro: simulation of an i.v. bolus and 3 h and 20 h infusion.

**DOI:** 10.1038/bjc.1994.166

**Published:** 1994-05

**Authors:** J. Ma, J. Verweij, H. J. Kolker, H. E. van Ingen, G. Stoter, J. H. Schellens

**Affiliations:** Department of Medical Oncology, Dr Daniel den Hoed Kliniek, Rotterdam, The Netherlands.

## Abstract

The profiles of an i.v. bolus and 3 h and 20 h infusion of cisplatin (CDDP) were simulated in vitro by using a culture of the IGROV1 human ovarian cancer cell line. Disappearance of pharmacologically active unbound CDDP was accomplished by adding human albumin to the medium. Total and unbound CDDP and CDDP-DNA adduct levels were quantitated by atomic absorption spectroscopy (AAS), and tumour cell survival was measured by the clonogenic assay. The design of the experiment resulted in non-significant differences in the magnitude of the area under the concentration-time curve (AUC) of unbound CDDP between the three dose-input functions (AUC i.v. bolus, 6.34 +/- 0.36; 3 h infusion, 6.35 +/- 0.59; and 20 h infusion, 6.76 +/- 0.40 micrograms h ml-1). Also, the differences between the area under the CDDP-DNA adduct-time curves (AUA) of the three dose-input functions were not significant. The initial rate of decline of the CDDP-DNA adduct-time curve was significantly higher for the i.v. bolus and 3 h infusion than for the 20 h infusion. There was a log-linear relationship between the AUC of unbound CDDP and cell survival. These relationships were not significantly different between the three dose-input functions. Variation in the rate of input of CDDP leads to differences in the shape of the AUC and AUA without significant effects on cell survival.


Br. J. Cancer (1994), 69, 858-862 © Macmillan Press Ltd., 1994

### Pharmacokinetic–dynamic relationship of cisplatin *in vitro*: simulation of an i.v. bolus and 3 h and 20 h infusion

J. Ma[1], J. Verweij[1], H.J. Kolker[1], H.E. van Ingen[2], G. Stoter[1] & J.H.M. Schellens[1]

[1]*Laboratory of Experimental Chemotherapy and Pharmacology, Department of Medical Oncology, and* [2]*Department of Clinical Chemistry, Rotterdam Cancer Institute, Dr Daniel den Hoed Kliniek, PO Box 5201, 3008 AE Rotterdam, The Netherlands.*

**Summary** The profiles of an i.v. bolus and 3 h and 20 h infusion of cisplatin (CDDP) were simulated *in vitro* by using a culture of the IGROV1 human ovarian cancer cell line. Disappearance of pharmacologically active unbound CDDP was accomplished by adding human albumin to the medium. Total and unbound CDDP and CDDP-DNA adduct levels were quantitated by atomic absorption spectroscopy (AAS), and tumour cell survival was measured by the clonogenic assay. The design of the experiment resulted in non-significant differences in the magnitude of the area under the concentration–time curve (AUC) of unbound CDDP between the three dose-input functions (AUC i.v. bolus, $6.34 \pm 0.36$; 3 h infusion, $6.35 \pm 0.59$; and 20 h infusion, $6.76 \pm 0.40\ \mu g\ h\ ml^{-1}$). Also, the differences between the area under the CDDP-DNA adduct–time curves (AUA) of the three dose-input functions were not significant. The initial rate of decline of the CDDP-DNA adduct–time curve was significantly higher for the i.v. bolus and 3 h infusion than for the 20 h infusion. There was a log-linear relationship between the AUC of unbound CDDP and cell survival. These relationships were not significantly different between the three dose-input functions. Variation in the rate of input of CDDP leads to differences in the shape of the AUC and AUA without significant effects on cell survival.

CDDP is one of the most potent cytotoxic compounds *in vivo* and is used frequently for the treatment of ovarian and testicular cancer, head and neck cancer and other malignancies (Loehrer & Einhorn, 1984; Forastiere *et al.*, 1987; Reed *et al.*, 1988*a*; 1990). Its application is accompanied by dose-limiting side-effects, such as nephro-, neuro- and ototoxicity (Kovacs *et al.*, 1982; Meijer *et al.*, 1983; Vermorken *et al.*, 1983). Despite its long-standing and wide clinical application, the optimal schedule of CDDP, with a maximal attainable anti-tumour effect and tolerable side-effects, has never been clearly established. From early studies it became evident that a rapid i.v. infusion was associated with the development of profound, and sometimes irreversible, nephrotoxicity. Because of this infusion times were prolonged, with intensive pre- and post-hydration and administration of CDDP in saline solutions (Jacobs *et al.*, 1978; Salem *et al.*, 1984; Vogelzang, 1984), rendering side-effects more manageable. The influence of the variation of the rate of input (i.e. dose-input function per treatment cycle) on the cytotoxic activity of CDDP is less clearly defined. Clinical observations suggest that the anti-tumour effect is not influenced by the dose-input function (Vermorken *et al.*, 1982).

Data on the pharmacokinetic–dynamic relationships of CDDP in clinical and preclinical studies, taking the CDDP-DNA adduct formation and repair and exposure to unbound CDDP into consideration, are lacking.

CDDP binds almost irreversibly to plasma and cellular components (Yotsuyanagi *et al.*, 1991). This results in an extremely long retention of CDDP in tissues (Loehrer & Einhorn, 1984). The elimination half-life of the pharmacologically active unbound CDDP in the plasma is short and is approximately 1 h in man (Vermorken *et al.*, 1982, 1986). The cytotoxic activity of CDDP is very likely correlated to the covalent binding to DNA, so-called inter- and intrastrand adduct formation (Plooy *et al.*, 1984). This is not an irreversible process because of the cellular capacity to remove the formed adducts (DNA repair) (Plooy *et al.*, 1984; Fichtinger-Schepman *et al.*, 1987).

Variation in the dose-input function results in a different shape of the plasma concentration–time curve of unbound CDDP. Because of the diffusion of CDDP to tumour tissues, this will also result in different concentration–time curves at the site of action. If one assumes linear pharmacokinetics, i.e. the magnitude of the AUC is not dependent on the dose-input function, then variation in the dose-input function will only lead to differences in the shape of the AUC.

In the present study the kinetics of CDDP-DNA adduct formation and repair was studied *in vitro* as a function of the variation in the dose-input of CDDP, using a well-characterised IGROV1 ovarian cancer cell line. The profiles of an i.v. bolus and 3 h and 20 h infusion were simulated *in vitro* and the pharmacokinetics of unbound CDDP was correlated to the CDDP-DNA adduct formation and repair and tumour cell survival (i.e. pharmacodynamics).

### Materials and methods

### *Chemicals*

Roswell Park Memorial Institute (RPMI) 1640 medium was obtained from Brunschwig (Amsterdam, The Netherlands), bovine calf serum (BCS) from Hyclone (Logan, UT, USA), dimethylsulphoxide (DMSO) and platin (Pt) standard solution (500 p.p.m.) from Baker (Deventer, The Netherlands), phosphate-buffered saline (PBS) from Boom (Meppel, The Netherlands) and insulin Neerlandicum from Organon (Oss, The Netherlands). Streptomycin, penicillin, gentamycin, glutamine and trypsin were obtained from Gibco (Breda, The Netherlands), DNAse I from Sigma (St Louis, MO, USA), proteinase K and caesium chloride from Merck (Darmstadt, Germany), and sodium dodecyl sulphate (SDS) and haematoxylin from Brunschwig (Amsterdam, The Netherlands). Ethylenediaminetetraacetic acid (EDTA) and all other chemicals were obtained from Baker and were of analytical grade or higher. T25 ($25\ cm^2$), T75 ($75\ cm^2$) and T175 ($162\ cm^2$) culture flasks were obtained from Costar (Badhoevedorp, The Netherlands).

### *IGROV1 cell culture*

The IGROV1 ovarian adenocarcinoma cell line was originated by J. Bénard (Institut Gustave Roussy, Villejuif, France (Bénard *et al.*, 1985; Teyssier *et al.*, 1989) and kindly supplied by R.L.H. Bolhuis (Rotterdam Cancer Institute, Rotterdam, The Netherlands). The cell line was maintained in a continuous logarithmic culture in RPMI-1640 medium

Correspondence: J.H.M. Schellens.
Received 27 August 1993; and in revised form 20 December 1993.

with HEPES and phenol red supplemented with 10% BCS, 10 mM sodium bicarbonate, 2 mM glutamine, penicillin 111 IU $ml^{-1}$, streptomycin 103 $\mu g\ ml^{-1}$, gentamycin 43 $\mu g\ ml^{-1}$ and insulin 10 $\mu g\ ml^{-1}$. The cells were cultured at 37°C in a humidified atmosphere of 5% carbon dioxide in air. The cells were mildly trypsinised for passage and for use in experiments. The cloning efficacy and cell doubling time were determined. For the latter the sulphorhodamine B (SRB) test was used.

*Apparatus*

A flameless Perkin-Elmer 3030B atomic absorption spectrophotometer (AAS) was used equipped with an AS60 autosampler and HGA600 controller system (Überlingen, Germany). The UV spectrophotometer was a Backman DU62 (Fullerton, CA, USA) set at 260 nm.

*Assay of CDDP in the culture medium*

Total and unbound CDDP were analysed by AAS. One millilitre of medium was taken for the analysis of the total and unbound CDDP concentration. For the total CDDP concentration, 100 $\mu l$ was taken and diluted 10–40 times with 0.2% Triton X-100 containing 0.06% caesium chloride in order to obtain CDDP concentrations in the range 10–100 ng $ml^{-1}$. Unbound CDDP was analysed after deproteination of 0.5 ml of the medium sample with 1.0 ml of ice-cold absolute ethanol. A volume of 250 $\mu l$ was taken and diluted 10–40 times with deionised water. A volume of 50 $\mu l$ was injected into the AAS. All measurements were carried out in duplicate.

*Assay of DNA levels in tumour cells*

DNA levels in tumour cells were determined according to a method described by Fichtinger-Schepman *et al.* (1987).

*Analysis of CDDP-DNA adduct levels*

CDDP-DNA adduct levels were quantitated according to a method described by Reed *et al.* (1988*b*) with modifications. Briefly, a DNA sample was digested with DNAse I and zinc chloride (10 $\mu$M, 10 $\mu l$) was added to the mixture to optimise the enzymatic reaction (Fichtinger-Schepman *et al.*, 1987). A volume of 160 $\mu l$ (approximately 60–250 $\mu g$ of DNA) was injected into the furnace using the 4 × multiple sampling feature of the instrument. The samples were calibrated on a standard curve of four samples with a Pt concentration of 0, 1.5, 3, 6 ng $ml^{-1}$ (equivalent to 0, 240, 480, 960 pg of Pt in the injected volume of 160 $\mu l$). CDDP-DNA adduct levels were expressed as pg of Pt per $\mu g$ of DNA (pg Pt $\mu g^{-1}$DNA). The analysis was carried out in duplicate.

*CDDP-protein binding experiment*

CDDP (10 $\mu g\ ml^{-1}$) was incubated for 24 h at 37°C with human plasma (15 ml) and a 7% (v/v) human albumin solution in RPMI-1640, containing 10% BCS (total volume of the mixture, 15 ml). During the incubation period total and unbound CDDP were analysed at 0, 0.5, 1, 2, 3, 5, 8 and 24 h.

*Influence of protein binding on the CDDP-DNA adduct formation*

In order to study the relationship between the unbound CDDP and adduct formation, IGROV1 cells were incubated with CDDP in three different experiments.

1. A volume of 5 ml of PBS was added to each of three T75 flasks containing the cell culture in 10 ml of RPMI-1640. Subsequently CDDP was added.
2. Instead of PBS, 5 ml of a 20% albumin solution was added, immediately followed by CDDP.
3. CDDP was preincubated with 5 ml of the 20% albumin solution at 37°C for 24 h and added to the cell culture. The final concentration of CDDP in each flask was 5 $\mu g\ ml^{-1}$. All experiments were carried out in triplicate. At 1, 2 and 4 h after the start of the incubation a flask was taken and analysed for total and unbound CDDP and the CDDP-DNA adduct level.

*Design of the simulation of the profiles of an i.v. bolus and 3 h and 20 h infusions*

*Intravenous bolus profile* Six T175 culture flasks containing approximately 6 × $10^6$ cells in 16 ml of medium were used for the concentration–time and adduct–time curves. An 8 ml aliquot of albumin was added immediately followed by 90 $\mu l$ of a CDDP solution of 1 mg $ml^{-1}$. The final albumin concentration in the mixture was then 7%. After 3 h the medium was carefully removed and the cells were washed twice with PBS and cultured again in CDDP-free medium. At 1, 3, 8, 20, 44 and 68 h from the start a flask was used to harvest cells for measurement of the CDDP-DNA adduct level. During the incubation 1 ml of medium was taken at 0, 0.5, 1, 2 and 3 h for measurement of the concentration of total and unbound CDDP.

*Three hour infusion profile* Every 15 min for 1½ h a small, but constant, amount of CDDP (18.4 $\mu l$ containing 0.2 mg $ml^{-1}$ CDDP) was added to the 16 ml of culture medium. After another 1½ h of incubation 8 ml of 20% albumin was added to the flask immediately followed by CDDP (51.2 $\mu l$ of a solution of 0.2 mg $ml^{-1}$ CDDP), to prevent dilution of CDDP in the flask owing to the addition of the relatively large volume of albumin solution. Five hours later (8 h from the start) the medium was removed and the cells were washed and cultured again as outlined. The same CDDP-DNA adduct time points were determined as in the i.v. bolus experiment. During the incubation 1 ml of medium was taken at 0, 0.5, 1, 2, 3 and 8 h for measurement of the total and unbound CDDP concentration.

*Twenty-hour infusion profile* Seven flasks were used. CDDP was added at time point 0 h, and after 20 h the medium was removed. Subsequently, the cells were washed and cultured as outlined. The CDDP-DNA adduct time points were 1, 3, 8, 20, 24, 44 and 68 h. Total and unbound CDDP were determined at 1, 3, 8 and 20 h, as outlined. All experiments are carried out in quadruplicate. The three experiments were further denoted as i.v. bolus and 3 h and 20 h infusions.

*Cell survival measurement*

The clonogenic assay was used. Exponentially growing IGROV1 cells in RPMI-1640 were plated in T25 culture flasks (in 4 ml of medium), with a density range of 5 × $10^2$ to $10^5$ cells, 24 h before CDDP administration. The same procedure of CDDP incubation and albumin addition was followed as outlined above. In the i.v. bolus experiment individual flasks were taken after 1, 2 and 3 h of incubation, in the 3 h infusion experiment after 1, 3 and 8 h of incubation, and in the 20 h infusion after 1, 3, 8 and 20 h of incubation. The medium was removed and the cells were washed twice with PBS and cultured again in CDDP-free medium for approximately 2 weeks. Subsequently, the colonies were fixed with absolute methanol–acetic acid (17 M) (2:1, v/v) and stained with haematoxylin. Colonies of $>50$ cells were counted. All experiments were carried out in quadruplicate.

*Pharmacokinetic and statistical analysis*

The AUC of unbound CDDP in the medium and AUA (up to 68 h) in the tumour cells were calculated using the trapezoidal method. The initial slope, after withdrawal of CDDP, was calculated between the adduct time points 3–20 h (i.v. bolus, three adduct time points), 8–20 h (3 h infusion, two points) and 20–44 h (20 h infusion, three points). Student's *t*-test, the Kolmogorov–Smirnov (KS) test and log-linear regression analysis were used. Student's $t$-test was used if at least four observations were evaluable and the standard deviations per treatment group were <400% different, otherwise the non-parametric KS test was applied.

### Results

### *Igrov1 cell culture (IGROV1)*

The cloning efficacy was 20 ± 4%. The cell doubling time was 24 ± 3 h.

### *CDDP-protein binding experiment*

The binding of CDDP to proteins in human plasma was similar to the binding to proteins in the mixture of RPMI-1640 containing human albumin (Figure 1). After 1 h approximately 50% of CDDP was bound in both experiments. After 24 h only 3% was unbound.

In the protein-free medium there was a linear increase in adduct formation with time (Figure 2). Addition of albumin, immediately followed by CDDP, resulted in a significantly lower increase in adducts with time. When CDDP was preincubated with albumin, thereby eliminating unbound CDDP, no significant adduct formation was observed.

### *Assay of CDDP in the culture medium*

Total and unbound CDDP could be analysed reproducibly in the observed concentration range 0.1–5 $\mu g\ ml^{-1}$. Coefficients of variation were <5%. The measured concentration–time curves of total and unbound CDDP in the three experiments are given in Figure 3. The profiles of unbound CDDP in the simulated i.v. bolus and 3 h and 20 h infusions were not significantly different from those expected in plasma. The elimination half-life of unbound CDDP in the i.v. bolus experiment was 1.5 ± 0.20 h, and in the 3 h infusion experiment 1.7 ± 0.20 h (not significantly different). In the i.v. bolus experiment only 22% of CDDP was unbound after 3 h of incubation. In the 20 h infusion experiment 55% was unbound after 20 h of incubation (Figure 3). The $C_{max}$ of CDDP was highest in the i.v. bolus and lowest in the 20 h infusion experiment (Table I). The AUC values of the three dose-input functions were not significantly different.

### *Analysis of DNA and CDDP-DNA adduct levels*

CDDP-DNA adduct levels could be determined reproducibly in the observed range 0.5–15 pg Pt $\mu g^{-1}$DNA. The coefficient of variation was 20% at 1.5 ng $ml^{-1}$Pt (240 pg of Pt per injected sample) and 8% at higher concentrations. The detection limit was 100 pg of Pt per sample.

The maximal adduct levels ($A_{max}$) were reached at the end of the incubation with CDDP (Figure 4). The differences between the three experiments were not significant (Table I), although there appeared to be a rank order, with the i.v.

**Figure 2** Relationship between the exposure of CDDP (5 $\mu g\ ml^{-1}$) and CDDP-DNA adduct levels in the IGROV1 cell line. **a**, CDDP in RPMI-1640 medium with 6 × $10^6$ cells. **b**, CDDP in a 7% solution of human albumin in RPMI-1640. **c**, CDDP was preincubated with human albumin for 24 h and added to RPMI-1640. *$P$<0.05; **$P$<0.01 (**a** compared with **b**); ***$P$<0.01 (**c** compared with **b**). NS, not significant.

**Figure 1** Protein binding of CDDP (10 $\mu g\ ml^{-1}$) in human plasma (O) and in a 7% solution of human albumin in RPMI-1640 (+). Incubation at 37°C.

**Figure 3** Total and unbound CDDP of the three dose-input functions. ▲, △, total and unbound CDDP of the i.v. bolus; ●, O, total and unbound CDDP of the 3 h infusion; ■, □, total and unbound CDDP of the 20 h infusion.

**Table I** Pharmacokinetic and dynamic data of CDDP *in vitro* (mean ± s.d. of four observations)

| Dose-input | $C_{max}$ ($\mu g\ ml^{-1}$) | AUC ($\mu g\ h\ ml^{-1}$) | $A_{max}$ (pg Pt $\mu g^{-1}$ DNA) | AUC (pg Pt h $\mu g^{-1}$ DNA) | Initial slope (%) |
|---|---|---|---|---|---|
| Intravenous bolus | 4.23 ± 0.12[a] | 6.33 ± 0.36 | 8.8 ± 0.9 | 293 ± 32 | 21 ± 1[b,c] |
| Three hour infusion | 1.47 ± 0.03 | 6.34 ± 0.59 | 8.5 ± 0.7 | 271 ± 10 | 31 ± 9[b] |
| Twenty hour infusion | 0.52 ± 0.05 | 6.76 ± 0.40 | 7.4 ± 1.2 | 319 ± 49 | 13 ± 1 |
| | | NS | NS | NS | |

[a]$P$<0.05: 0.52<1.47<4.23, [b]$P$<0.05 compared with 20 h infusion, [c]$P$ = 0.07 compared with 3 h infusion. NS, not significantly different.

**Figure 4** CDDP-DNA adduct-time curves of the three dose-input functions. (+) Intravenous bolus; (O) 3 h infusion; (●) 20 h infusion. *$P<0.01$: i.v. bolus significantly higher than 3 h infusion and 3 h infusion significantly higher than 20 h infusion. **$P<0.01$: i.v. bolus and 3 h infusion significantly higher than 20 h infusion. ***$P<0.01$: 20 h infusion significantly higher than i.v. bolus and 3 h infusion. ****$P<0.05$: 20 h infusion significantly higher than i.v. bolus and 3 h infusion.

**Figure 5** Influence of the three dose-input functions of CDDP on cell survival of IGROV1 tumour cells in the clonogenic assay. (+) Intravenous bolus; (O) 3 h infusion; (●) 20 h infusion. *$P<0.01$: 20 h infusion significantly higher than 3 h infusion and 3 h infusion significantly higher than i.v. bolus. **$P<0.05$: 20 h infusion significantly higher than 3 h infusion.

bolus having the highest and the 20 h infusion experiment the lowest $A_{max}$ level. Also, the AUA values, calculated up to 68 h, were not significantly different between the experiments. The initial decline in the adduct-time curve (initial slope in Table I) was significantly more rapid in the i.v. bolus and 3 h infusion than in the 20 h infusion experiment.

*Cell survival measurement*

The decline in cell survival with time was highest in the i.v. bolus and lowest in the 20 h infusion experiment (Figure 5). A log-linear relationship was observed between the decline in the cell survival and the AUC of unbound CDDP (Figure 6). The slopes of the three dose-input functions were not significantly different (Figure 6). The log-linear slope of the i.v. bolus experiment was 12.9 ± 0.13, of the 3 h experiment 13.2 ± 0.66 and of the 20 h experiment 11.7 ± 0.53 (all $n = 3$; not significantly different).

**Figure 6** Log-linear relationship between the AUC of unbound CDDP of the three dose-input functions of CDDP and cell survival. (+) Intravenous bolus; (O) 3 h infusion; (●) 20 h infusion.

### Discussion

In the present study relationships were established between the exposure to unbound CDDP, CDDP-DNA adduct kinetics and tumour cell survival. To assess these relationships former and presently used clinical dose-input functions were simulated *in vitro*, using a cell culture of the IGROV1 human ovarian cancer cell line.

After administration to patients, unbound CDDP is eliminated by irreversible protein binding and renal elimination. The clearance by protein binding exceeds renal clearance of unbound CDDP by a factor of approximately 4–5 (Bajorin *et al.*, 1986). Irreversible protein binding can easily be simulated *in vitro*, as is illustrated in Figure 1. Human albumin was added to the RPMI-1640 culture medium of the IGROV1 cell line as outlined. The results show that the protein binding kinetics in this mixture and in human plasma are similar. The concentration range of CDDP *in vitro* in the present study was of the same order as is observed in plasma of patients after administration of a dose of approximately 80 mg m$^{-2}$ (Vermorken *et al.*, 1986). The elimination kinetics of CDDP *in vitro*, using the outlined approach, reflects the kinetics *in vivo* (Vermorken *et al.*, 1982, 1986). The elimination half-life is slightly longer because of the absence of renal clearance. In the present model the remaining low concentration of unbound CDDP was removed from the medium after 3, 8 and 20 h in the i.v. bolus, 3 h and 20 h experiment respectively, to prevent low remaining concentrations of unbound CDDP and to facilitate the calculation of the AUC.

The simulation of the clinical dose-input functions of CDDP *in vitro* has some limitations. The cell line is exposed to concentration–time profiles of CDDP which are observed *in vivo* in the blood compartment. This profile may be different from the *in vivo* exposure of tumour cells in solid tumours in peripheral tissues in patients. The concentration of unbound CDDP to which the tumour cells are exposed in the *in vitro* model will be slightly different from the concentration in the tumour in patients, because no steady state has been reached after a single CDDP administration. Another limitation of the model is associated with the use of a logarithmic growing cell culture instead of the use of a solid tumour. However, because of the similarities between the pharmacokinetics of unbound CDDP *in vivo* in patients and *in vitro* in the present model, the model is considered appropriate for the assessment of pharmacokinetic–dynamic relationships of unbound CDDP, despite the outlined restrictions.

Protein-bound CDDP is not able to form CDDP-DNA adducts, which is illustrated in Figure 2. Hence unbound CDDP should be used for the assessment of the pharmacokinetic–dynamic relationship. In the light of this observation it should be realised that clinical trails which use CDDP

preincubated with albumin (Holding et al., 1992) may not lead to significant anti-tumour responses.

The design of the simulation of the three dose-input functions resulted in non-significant differences in the AUC of unbound CDDP. This is an ideal starting point to assess the influence of the rate of input of CDDP on CDDP-DNA adduct formation and repair and cell survival.

The $A_{max}$ in the three experiments was not significantly different, although the level in the i.v. bolus and 3 h infusion was slightly higher than in the 20 h infusion. The AUA was clearly not significantly different in the three dose-input functions. This illustrates that the rate of input of CDDP does not influence the magnitude of the adduct formation.

The initial rate of decline of the adduct-time curve was significantly lower in the 20 h infusion. The rate of decline was studied in all experiments after withdrawal of CDDP. It is not clear what mechanism caused the difference. Furthermore, the increase in the adduct formation with time in the 20 h infusion experiment is clearly non-linear (Figure 4). However, the experimental design does not permit assessment of differences in adduct removal during the period of CDDP exposure. The observed differences in the adduct kinetics had no implications for cell survival (Figure 6). This seems to be in contrast to the results of the Troger et al. (1992). They found that the longest exposure time of total CDDP in a head and neck cancer cell line resulted in the lowest cytotoxicity. However, protein binding of CDDP in the culture medium was not taken into account and no adduct levels were determined. Correction for the unavoidable protein binding of CDDP might have changed the results of that study.

The log-linear relationship between the AUC of unbound CDDP and cell survival was strikingly similar in the three dose-input functions. This implies that cell kill is determined not by the rate of input of CDDP, but by the magnitude of the exposure to unbound CDDP. If these results are confirmed in other tumour models, then they may have significant implications in designing optimal modes of clinical CDDP administration.

We thank Dr A.M.J. Fichtinger-Schepman, MBL-TNO The Netherlands, for her assistance in the validation of the CDDP-DNA adduct assay and Dr K. Nooter, Department of Medical Oncology, Rotterdam Cancer Institute/Academic Hospital Rotterdam, The Netherlands, for critically reading the manuscript.

### References

BAJORIN, D.F., BOSL, G.J., ALCOCK, N.W., NIEDZWIECKI, D., GALLIA, E. & SHURGOT, B. (1986). Pharmacokinetics of cis-diamminedichloroplatinum (II) after administration in hypertonic saline. *Cancer Res.*, **46**, 5969-5972.

BENARD, J., DA SILVA, J., DE BLOIS, M.-C., BOYER, P., DUVILLARD, P., CHIRIC, E. & RIOU, G. (1985). Characterization of a human ovarian adenocarcinoma line, IGROV1, in tissue culture and in nude mice. *Cancer Res.*, **45**, 4970-4979.

FICHTINGER-SCHEPMAN, A.M.J., VAN OOSTEROM, A.T., LOHMAN, P.H.M. & BERENDS, F. (1987). cis-Diamminedichloroplatinum-induced DNA adducts in peripheral leukocytes from seven cancer patients: quantitative immunochemical detection of the adduct induction and removal after a single dose of cis-diamminedichloroplatinum (II). *Cancer Res.*, **47**, 3000-3004.

FORASTIERE, A.A., TAKASUGI, B.J., BAKER, S.R., WOLF, G.T. & KUDLA-HATCH, V. (1987). High-dose cisplatin in advanced head and neck cancer. *Cancer Chemother. Pharmacol.*, **19**, 155-158.

HOLDING, J.D., LINDUP, W.E., VAN LAER, C., VREEBURG, G.C.M., SCHILLING, V., WILSON, J.A. & STELL, P.M. (1992). Phase I trial of a cisplatin-albumin complex for the treatment of cancer of the head and neck. *Br. J. Clin. Pharmacol.*, **33**, 75-81.

JACOBS, C., BERTINO, J.R., GOFFINET, D.R., FEE, W.R. & GOODE, R.L. (1978). 24-hour infusion of cis-platinum in head and neck cancers. *Cancer*, **42**, 2135-2140.

KOVACS, C.J., BRAUNSCHWEIGER, P.G., SCHENKEN, L.L. & BURHOLT, D.R. (1982). Proliferative defects in renal and intestinal epithelium after cis-dichlorodiammineplatinum (II). *Br. J. Cancer*, **45**, 286-294.

LOEHRER, P.J. & EINHORN, L.H. (1984). Diagnosis and treatment. Drugs five years later. *Ann. Intern. Med.*, **100**, 704-713.

MEIJER, S., MULDER, N.H. & SLEIJFER, D.T. (1983). Influence of combination therapy with cis-diamminedichloroplatinum (II) on renal function: long-term effects. *Oncology*, **40**, 170-173.

PLOOY, A.C.M., VAN DIJK, M. & LOHMAN, P.H.M. (1984). Induction and repair of DNA cross-links in chinese hamster ovary cells treated with various platinum coordination compounds in relation to platinum binding to DNA cytotoxicity, mutagenicity and antitumour activity. *Cancer Res.*, **44**, 2043-2051.

REED, E., OZOLS, R.F., TARONE, R., YUSPA, S.H. & POIRIER, M.C. (1988*a*). The measurements of cisDDP-DNA adduct levels in testicular cancer patients. *Carcinogenesis*, **9**, 1909-1911.

REED, E., SAUERHOFF, S. & POIRIER, M.C. (1988*b*). Quantitation of platinum-DNA binding after therapeutic levels of drug exposure - a novel use of graphite furnace spectrometry. *Atomic Spectroscopy*, **9**, 93-95.

REED, E., OSTCHEGA, Y., STEINBERG, S.M., YUSPA, S.H., YOUNG, R.C., OZOLS, R.F. & POIRIER, M.C. (1990). Evaluation of platinum-DNA adduct levels relative to known prognostic variables in a cohort of ovarian cancer patients. *Cancer Res.*, **50**, 2256-2260.

SALEM, P., KAHLYL, M., JABBOURY, K. & HASHIMI, L. (1984). Cis-diamminedichloroplatinum (II) by 5-day continuous infusion. A new dose schedule with minimal toxicity. *Cancer*, **53**, 837-840.

TEYSSIER, J.R., BENARD, J., FERRE, D., DA SILVA, J., RENAUD, L. (1989). Drug-related chromosomal changes in chemoresistant human ovarian. *Cancer Genet. Cytogenet*, **39**, 35-43.

TROGER, V., FISCHEL, J.L., FORMENTO, P., GIOANNI, J. & MILANO, G. (1992). Effects of prolonged exposure to cisplatin on cytotoxicity and intracellular drug concentration. *Eur. J. Cancer*, **28**, 82-86.

VERMORKEN, J.B., VAN DER VIJGH, W.J.F., KLEIN, I., GALL, H.E. & PINEDO, H.M. (1982). Pharmacokinetics of free platinum species following rapid, 3 h and 24 h infusions of cis-diamminedichloroplatinum (II) and its therapeutic implications. *Eur. J. Cancer Clin. Oncol.*, **18**, 1069-1074.

VERMORKEN, J.B., KAPTEIJN, T.S., HART, A.A.M. & PINEDO, H.M. (1983). Ototoxicity of cis-diamminedichloroplatinum (II). Influence of dose, schedule and mode of administration. *Eur. J. Cancer Clin. Oncol.*, **19**, 53-58.

VERMORKEN, J.B., VAN DER VIJGH, W.J.F., KLEIN, I., GALL, H.E., VAN GROENINGEN, C.J., HART, A.A.M. & PINEDO, H.M. (1986). Pharmacokinetics of free and total platinum species following rapid and prolonged infusions of cisplatin. *Clin. Pharmacol. Ther.*, **39**, 136-144.

VOGELZANG, N.J. (1984). Continuous infusion chemotherapy: a critical review. *J. Clin. Oncol.*, **2**, 1289-1304.

YOTSUYANAGI, T., OHTA, N., FUTO, T., ITO, S., CHEN, D. & IKEDA, K. (1991). Multiple and irreversible binding of cis-diamminedichloroplatinum(II) to human serum albumin and its effect on warfarin binding. *Chem. Pharm. Bull.*, **39**, 3003-3006.


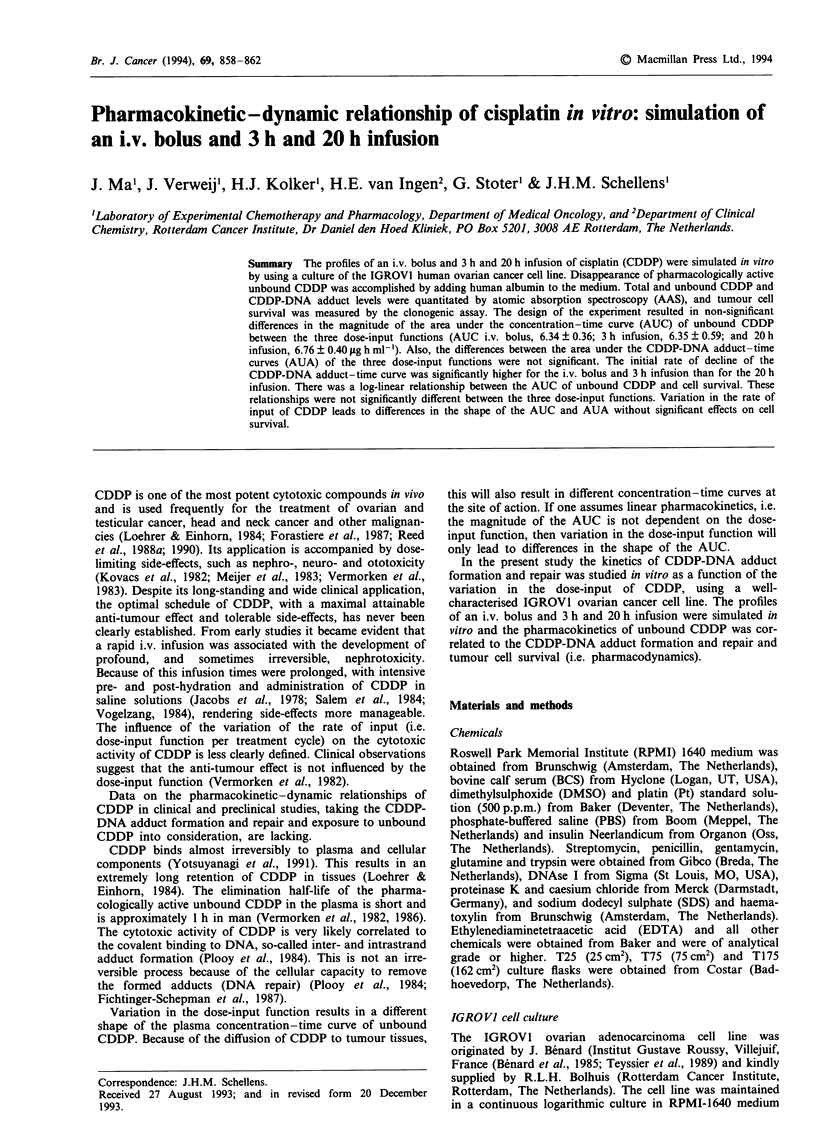

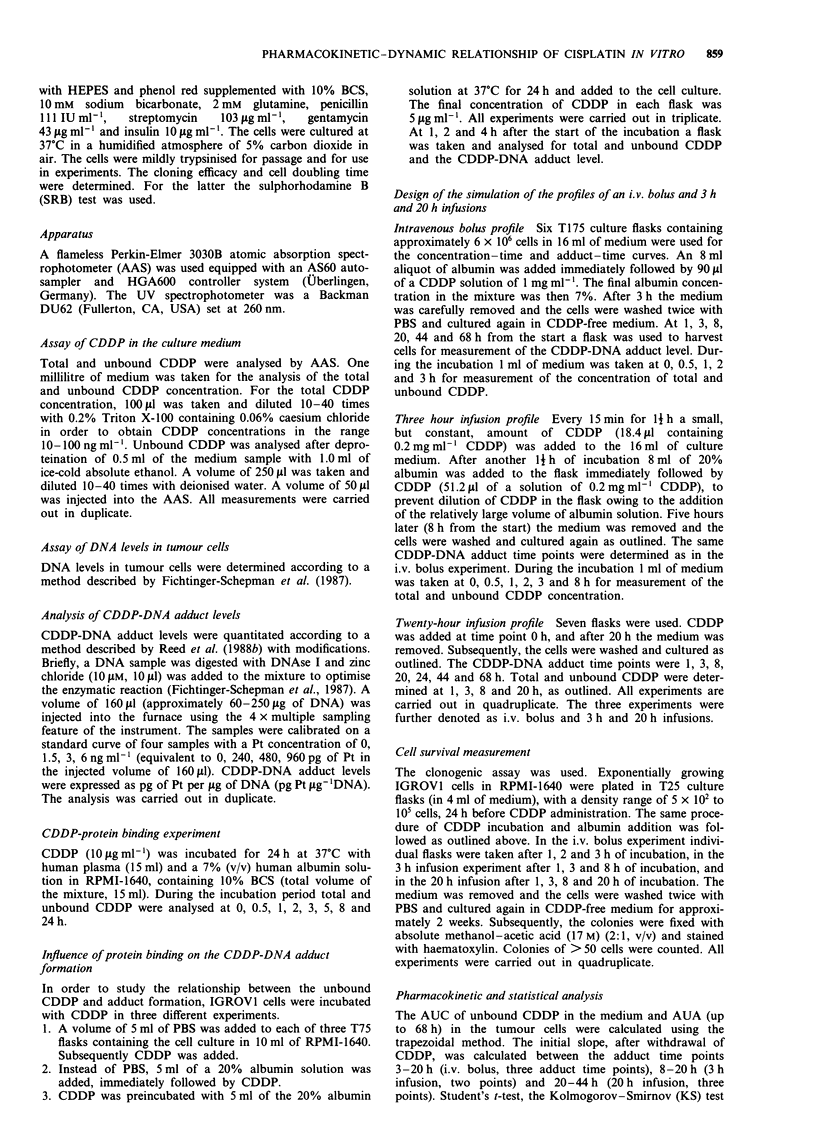

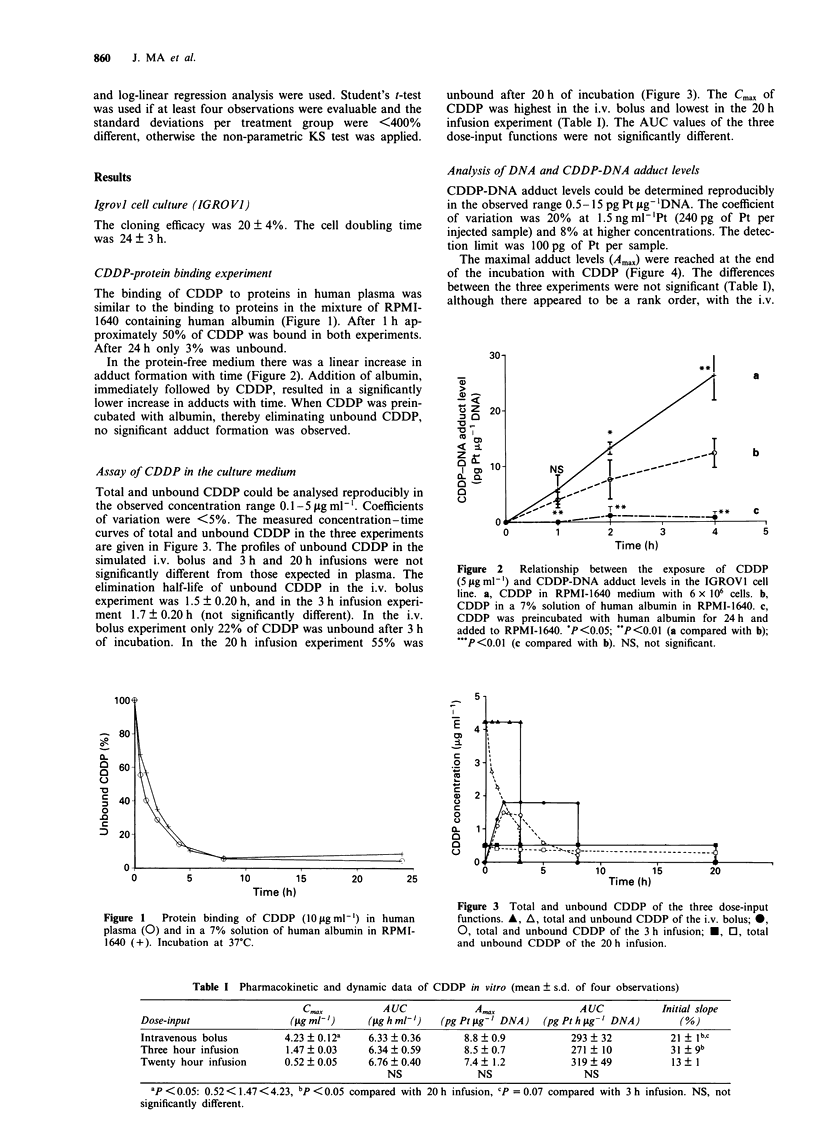

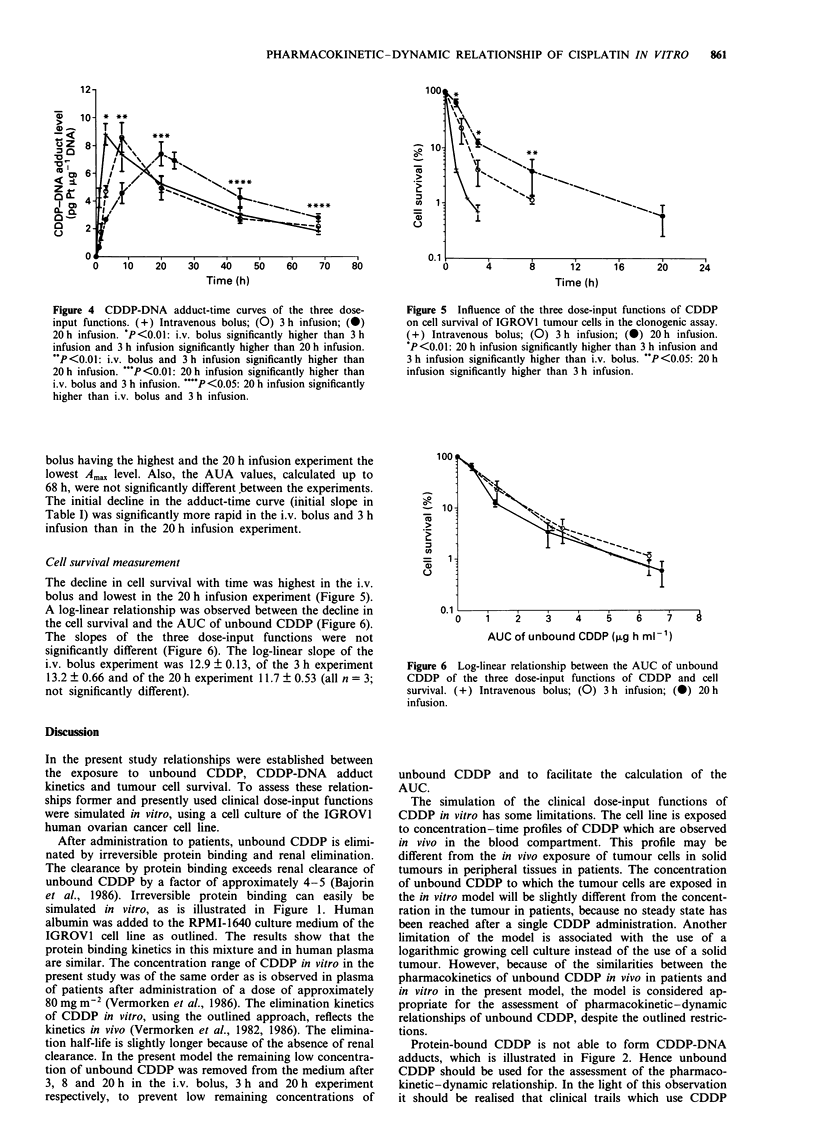

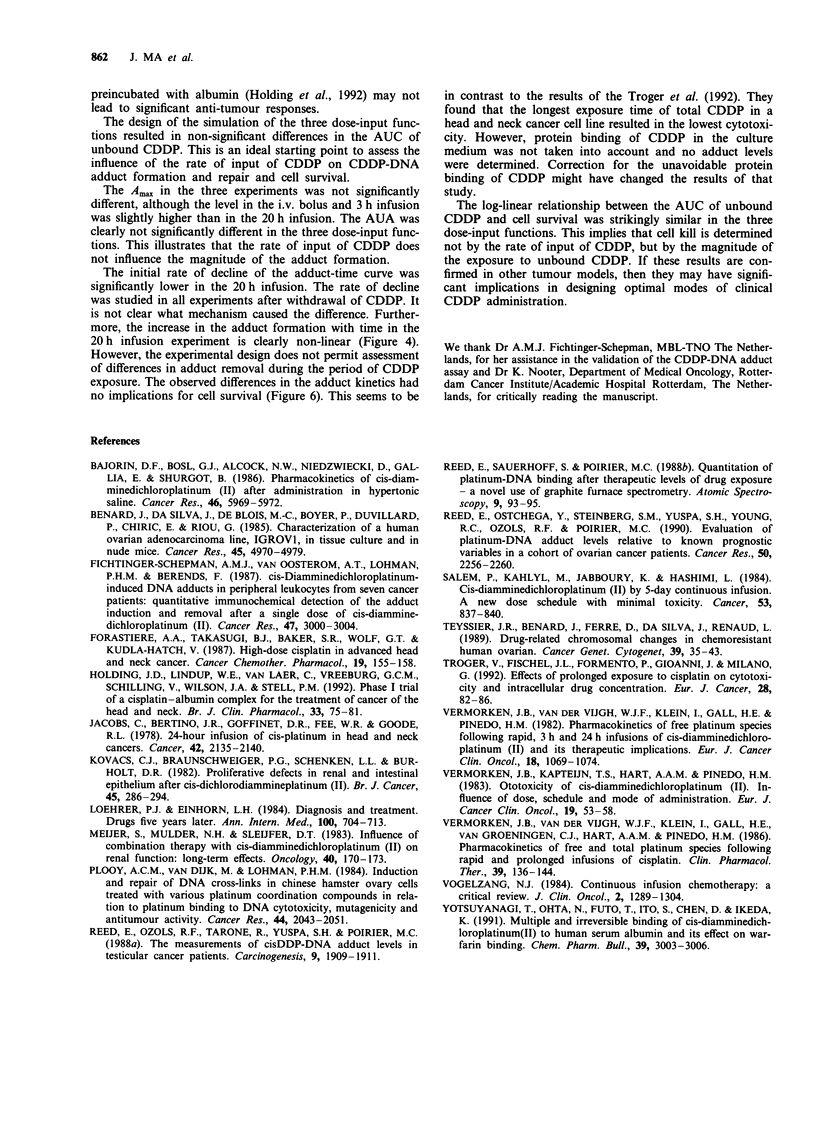


## References

[OCR_00629] Bajorin D. F., Bosl G. J., Alcock N. W., Niedzwiecki D., Gallina E., Shurgot B. (1986). Pharmacokinetics of cis-diamminedichloroplatinum(II) after administration in hypertonic saline.. Cancer Res.

[OCR_00633] Bénard J., Da Silva J., De Blois M. C., Boyer P., Duvillard P., Chiric E., Riou G. (1985). Characterization of a human ovarian adenocarcinoma line, IGROV1, in tissue culture and in nude mice.. Cancer Res.

[OCR_00639] Fichtinger-Schepman A. M., van Oosterom A. T., Lohman P. H., Berends F. (1987). cis-Diamminedichloroplatinum(II)-induced DNA adducts in peripheral leukocytes from seven cancer patients: quantitative immunochemical detection of the adduct induction and removal after a single dose of cis-diamminedichloroplatinum(II).. Cancer Res.

[OCR_00647] Forastiere A. A., Takasugi B. J., Baker S. R., Wolf G. T., Kudla-Hatch V. (1987). High-dose cisplatin in advanced head and neck cancer.. Cancer Chemother Pharmacol.

[OCR_00651] Holding J. D., Lindup W. E., van Laer C., Vreeburg G. C., Schilling V., Wilson J. A., Stell P. M. (1992). Phase I trial of a cisplatin-albumin complex for the treatment of cancer of the head and neck.. Br J Clin Pharmacol.

[OCR_00657] Jacobs C., Bertino J. R., Goffinet D. R., Fee W. E., Goode R. L. (1978). 24-hour infusion of cis-platinum in head and neck cancers.. Cancer.

[OCR_00664] Kovacs C. J., Braunschweiger P. G., Schenken L. L., Burholt D. R. (1982). Proliferative defects in renal and intestinal epithelium after cis-dichlorodiammine platinum (II).. Br J Cancer.

[OCR_00668] Loehrer P. J., Einhorn L. H. (1984). Drugs five years later. Cisplatin.. Ann Intern Med.

[OCR_00672] Meijer S., Mulder N. H., Sleijfer D. T., Donker A. J., Sluiter W. J., de Jong P. E., Schraffordt Koops H., van der Hem G. K. (1983). Influence of combination chemotherapy with cis-diamminedichloroplatinum on renal function: long-term effects.. Oncology.

[OCR_00677] Plooy A. C., van Dijk M., Lohman P. H. (1984). Induction and repair of DNA cross-links in chinese hamster ovary cells treated with various platinum coordination compounds in relation to platinum binding to DNA, cytotoxicity, mutagenicity, and antitumor activity.. Cancer Res.

[OCR_00695] Reed E., Ostchega Y., Steinberg S. M., Yuspa S. H., Young R. C., Ozols R. F., Poirier M. C. (1990). Evaluation of platinum-DNA adduct levels relative to known prognostic variables in a cohort of ovarian cancer patients.. Cancer Res.

[OCR_00684] Reed E., Ozols R. F., Tarone R., Yuspa S. H., Poirier M. C. (1988). The measurement of cisplatin-DNA adduct levels in testicular cancer patients.. Carcinogenesis.

[OCR_00702] Salem P., Khalyl M., Jabboury K., Hashimi L. (1984). Cis-diamminedichloroplatinum (II) by 5-day continuous infusion. A new dose schedule with minimal toxicity.. Cancer.

[OCR_00708] Teyssier J. R., Bénard J., Ferre D., Da Silva J., Renaud L. (1989). Drug-related chromosomal changes in chemoresistant human ovarian carcinoma cells.. Cancer Genet Cytogenet.

[OCR_00713] Troger V., Fischel J. L., Formento P., Gioanni J., Milano G. (1992). Effects of prolonged exposure to cisplatin on cytotoxicity and intracellular drug concentration.. Eur J Cancer.

[OCR_00726] Vermorken J. B., Kapteijn T. S., Hart A. A., Pinedo H. M. (1983). Ototoxicity of cis-diamminedichloroplatinum (II): influence of dose, schedule and mode of administration.. Eur J Cancer Clin Oncol.

[OCR_00719] Vermorken J. B., van der Vijgh W. J., Klein I., Gall H. E., Pinedo H. M. (1982). Pharmacokinetics of free platinum species following rapid, 3-hr and 24-hr infusions of cis-diamminedichloroplatinum (II) and its therapeutic implications.. Eur J Cancer Clin Oncol.

[OCR_00732] Vermorken J. B., van der Vijgh W. J., Klein I., Gall H. E., van Groeningen C. J., Hart G. A., Pinedo H. M. (1986). Pharmacokinetics of free and total platinum species after rapid and prolonged infusions of cisplatin.. Clin Pharmacol Ther.

[OCR_00739] Vogelzang N. J. (1984). Continuous infusion chemotherapy: a critical review.. J Clin Oncol.

[OCR_00743] Yotsuyanagi T., Ohta N., Futo T., Ito S., Chen D. N., Ikeda K. (1991). Multiple and irreversible binding of cis-diamminedichloroplatinum(II) to human serum albumin and its effect on warfarin binding.. Chem Pharm Bull (Tokyo).

